# Change in plasma lactate concentration during arctigenin administration in a phase I clinical trial in patients with gemcitabine-refractory pancreatic cancer

**DOI:** 10.1371/journal.pone.0198219

**Published:** 2018-06-01

**Authors:** Rumi Fujioka, Nobuo Mochizuki, Masafumi Ikeda, Akihiro Sato, Shogo Nomura, Satoshi Owada, Satoshi Yomoda, Katsuya Tsuchihara, Satoshi Kishino, Hiroyasu Esumi

**Affiliations:** 1 Division of Translational Research, Exploratory Oncology and Clinical Trial Center, National Cancer Center, Kashiwa, Japan; 2 Department of Medication Use Analysis and Clinical Research, Meiji Pharmaceutical University, Tokyo, Japan; 3 Department of Hepatobiliary and Pancreatic Oncology, National Cancer Center, Hospital East, Kashiwa, Japan; 4 Clinical Research Support Office, National Cancer Center Hospital East, Kashiwa, Japan; 5 Biostatistics Division, Center for Research Administration and Support, National Cancer Center, Kashiwa, Japan; 6 Research Institute for Biomedical Sciences, Tokyo University of Science, Noda, Japan; 7 Kanpo Research Institute, Kracie Pharmaceutical Company, Toyama, Japan; University of South Alabama Mitchell Cancer Institute, UNITED STATES

## Abstract

Arctigenin is evaluated for antitumor efficacy in patients with pancreatic cancer. It has an inhibitory activity on mitochondrial complex I.Therefore, plasma lactate level of patients after arctigenin administration was evaluated for biomarker of clinical response and/or adverse effect. Plasma lactate level in 15 patients enrolled in a Phase I clinical trial of GBS-01 rich in arctigenin was analyzed by colorimetric assay. Statistical analyses for association of plasma lactate and clinical responses, pharmacokinetics of arctigenin, and background factors of each patient by multivariate and univariate analyses.In about half of the patients, transient increase of lactate was observed. Correlation between plasma lactate level and pharmacokinetic parameters of arctigenin and its glucuronide conjugate, and clinical outcome was not detected. Regarding to the determinant of lactate level, only slight association with liver function test was detected. Plasma lactate level is primary determined by reutilization rather than production for antitumor effect and dose not serve as a biomarker. Arctigenin, inhibition of mitochondrial complex I, plasma lactate concentration, phase I clinical trial of GBS-01, Cori cycle.

## Introduction

Pancreatic cancer is one of the most refractory cancers. It is less symptomatic in the early stage and therefore hard to diagnose in the curable stage. The only curative method for the disease is surgery, but only one-sixth of newly diagnosed patients are indicative for surgery [1]. When the disease is treated by surgery, only a limited proportion of patients are cured. For patients with locally advanced inoperable stage and metastatic stage pancreatic cancer, chemotherapy such as molecular target therapy and radiotherapy provide only limited prognostic advantage, although remarkable advances in chemotherapy have recently been accomplished with FOLFIRINOX and nab-Paclitaxel [2]. It is thus desirable to develop a novel and effective treatment strategy for the disease.

Hypovascular feature is the most remarkable characteristics of human pancreatic cancer tissue. In analyzing the biology of pancreatic cancer cells, we observed characteristic tolerance to nutrient starvation *in vitro*, and involvement of the phosphatidylinositol 3-kinase (PI3K- AKT) pathway, and AMP-activated protein kinase (AMPK) activity in this tolerance, austerity [3, 4]. Under the nutrient-starved and severely hypoxic conditions that are thought to be due to hypovascularity of the cancer, a simple increase in glycolysis cannot be a mechanism for maintenance of energy states because of the limited glucose supply. We have reported that a very characteristic anaerobic adenosine triphosphate (ATP) generation system, fumarate respiration, may be operative [5]. In contrast to the hypovascularity of cancer tissues, long-lasting severely hypoxic and nutrient-starved conditions seldom occurs in normal tissue. We hypothesized that agents that would be toxic only under hypoxic and nutrient-starved conditions, antiausterity strategy would be useful for treating hypovascular cancers. We have developed a convenient screening system for antiausterity agents using the human pancreatic cancer cell line PANC-1 and identified several candidate compounds including kigamicin D, pyrvinium pamoate, angelmarin, and arctigenin (AG) [6–8].

AG is a natural lignan contained in many plants, including *Bardanae fructus*, *Ipomea cairica*, *Saussurea medusa*, and *Torreya nucifera*, and in the fruit of *Arctinum lappa* L., a Chinese traditional medicine. AG has lately received much attention for its wide variety of biological activities [9–11]. It has been shown to exert anti-inflammatory activity in many experimental systems [12]: neuroprotection, osteoclast inhibition, anti-arrhythmic effect, antidiabetic anti-hypercholesterolemia effects, and antitumor activity, [13, 14]. Because we have identified antiausterity activity of AG and antitumor activity in a human pancreatic cancer xenograft model [6] and the fruit of *A*. *lappa* appears in the Japanese Pharmacopoeia, we conducted a phase I clinical trial in patients with gemcitabine-refractory pancreatic cancer using an arctigenin-enriched extract of fruit of *A*. *lappa* (GBS-01). The results showed high bioavailability of AG after oral administration, a good safety profile, and promising clinical antitumor activity responses [15].

An extract of fruit of *A*. *lappa* has long been used as an anti-inflammatory agent in mastitis as a traditional medicine and the safety for this use has long been known. The biochemical and molecular targets of AG are unclear except that it inhibits the PI3K-AKT pathway during glucose starvation [6] and has recently been reported to inhibit mitochondrial complex I activity [16]. Actually we have recently found that the Reactive Oxygen Species (ROS) production is involved in the preferential cytotoxicity of AG during glucose starvation and ROS production is caused by inihibition of complex I by AG (Owada et al to be published).

Recently, compounds having inhibitory activity on mitochondrial respiration, including metformin, phenformin, and AG, have received much attention in cancer therapeutics [17]. When complex I activity is inhibited, cells produce increased amounts of lactate in response to increased glycolysis. Increased plasma lactate concentrations may cause adverse effects. It is thus important to understand the biochemical and pharmacological mechanisms of lactate production and the accumulation of lactate and its determinants. In addition, plasma lactate concentrations may be a pharmacodynamic biomarker of AG. We accordingly decided to examine plasma lactate profiles in patients in a phase I clinical trial of GBS-01.

## Patients and methods

### Patients

Fifteen patients were enrolled in the trial. The eligibility criteria for this study will be published elsewhere but are briefly as follows: cytologically or histologically proven invasive pancreatic ductal adenocarcinoma or adenosquamous carcinoma; refractoriness to gemcitabine-based chemotherapy; age ≧ 20 years; Eastern Cooperative Oncology Group (ECOG) performance status 0–2; adequate oral intake; satisfactory hematological functions (hemoglobin ≧ 8.5 g/dL, leukocytes ≧ 3,000 cells/mm^3^, neutrophils ≧1,500 cells/mm^3^, platelets ≧ 75,000/mm^3^); adequate hepatic function [serum total bilirubin ≦ 2.0 mg/dL or ≦ 3.0 mg/dL with biliary drainage, serum aspartate aminotransferase (AST) and alanine aminotransferase (ALT) ≦ 100 U/L or ≦ 150 U/L with biliary drainage]; adequate renal function [serum creatinine (Cr) ≦ 1.5 mg/dL]. Written informed consent was obtained from all patients [18].

### GBS-01

GBS-01 at dose level 1 (3.0 g/day) contains 2 g sucrose and burdock fruit extract 1.0 g, which in turn contains 68.5 mg of arctiin and 59.4 mg of AG (100 mg AG equivalent). The detailed method of preparation of GBS-01 is published elsewhere (Patent document JP-4963738-B2-). GBS-01 was given orally once daily after breakfast on consecutive days.

### Design of clinical trial

This was an open-label, single institutional, single-arm phase I study aimed at investigating the maximum tolerated dose of GBS-01 on the basis of the frequency of dose-limiting toxicities in patients with advanced PC refractory to gemcitabine (GEM) therapy. GBS-01 was administered orally once daily before breakfast every day until stopping criteria were fulfilled. Three dose levels were used: level 1, 3.0 g/day (containing 100 mg AG) was administered to three patients, level 2, 7.5 g/day (containing 250 mg AG) to three patients, and level 3, 12.0 g/day (containing 400 mg AG) to nine patients.

Blood sample for plasma AG measurements were drawn before and at 0.5, 1, 1.5, 2, 3, 4, 6, and 12 hours after administration of GBS-01 on days 1 and 8. The detailed method of determination of plasma AG and its glucuronide conjugate (AGG) will be published elsewhere. Residual plasma of patients enrolled in GBS-01 was used for plasma lactate analysis. Plasma lactate concentrations was determined by two methods: a lactate Pro2 sensor purchased from Arkley Co. and a lactate assay kit II purchased from BioVision (Miloitas).

This phase I study was conducted with the approval of the institutional review board of the National Cancer Center and in accordance with the Declaration of Helsinki and Ethical Guidelines for Clinical Research (Ministry of Health, Labour and Welfare, Japan). The trial was registered at UMIN-CTR (http://www.umin.ac.jp/ctr/index-j.htm) with identification number UMIN000005787. The Clinical Trial Section at the National Cancer Center Hospital East managed patient registrations and data collection. The quality of the data was ensured by a careful review performed by the staff of the data center and the coordinating investigator of this study (A.S., S.N., M.I.). All data were fixed on April 15, 2014 and analysis and interpretation of data was performed. (S.N., A.S., M.I., H.E.)

### Cell culture experiment

The human pancreatic cancer cell line PANC-1 obtained from the American Type Culture Collection, were maintained in Dulbecco's Modified Eagle Medium (DMEM) (Nissui, Tokyo, Japan) supplemented with 10% Fetal bovine serum (FBS) (biowest, Nusille, France), 2% L-glutamine, 1% antibiotics, and 1% MEN Non-Essential Amino Acids Solution (NEAA).

Lactate production in PANC-1 cell was measure of as follows; Cells were seeded at 8x10^4^ cells/well to 24 well plate and culture 24 hours. The culture media were collected at 0, 3, and 6 hours and lactate determination used for lactate assay kit II. Oxygen consumption rate (OCR) was examined with a Seahorse Extracellular Flux Analyzer model 24XF (Seahorse Bio Science, MA). Cells were seeded at 4 x 10^4^ cells/well in 24 well plates culture 24 hours before measurement. Effect of AG on mitochondrial respiration was examined by using XF Mito Stress Test Kit (Seahorse Bioscience, MA) according to the protocol recommended by the supplier.

### Statistical analysis

Univariate analyses were performed by regression analysis using general linear model to identify positive correlations between plasma lactate area under the curve (AUC) and AUC of AG or AGG, between lactate Cmax and AG Cmax or AGG Cmax, and correlations among plasma lactate AUC and WBC, white blood cell count; CRP, creactive protein; FBS, fasting blood sugar; ALB, serum albumin concentration; TBIL, total bilirubin; AST, aspartate transaminase; ALT, alanine transaminase; GGTP, gamma glutamyl transpeptidase; ALP, alkaline phosphatase; CRE, creatinine; CCr, creatinine clearance; and BUN, blood urea nitrogen by excel 2013.

To identify which renal and liver function variables had an influence on the plasma lactate Cmax and AUC, we performed multivariate regression analysis using general linear model. Association of each variable representing renal and liver function on the outcome parameter (Cmax and AUC) were examined by adjusting two possible confounding variables (surgical history and level of GBS-01 dose) in the general linear model. All P values were based on two-sided statistical tests, and this statistical analysis was done with SAS Release 9.3 (SAS Institute, Inc., Cary, NC).

When correlation between clinical responses and lactate level was analyzed, patients were divided into two groups depending on their lactate level, higher and lower than median. Progression free survival and overall survival time were compared btween two groups by Kaplan-Meier method. Lactate and clinical response was also analyzed by Student’s T test between mean values of Cmax or AUC of patients showed partial response (PR) or stable disease (SD) and progressive disease (PD). In all the statistical analyses, P value less than 0.05 was considered significant.

## Results

### AG treatment causes a large lactate increase in tissue culture

**Fig 1:**
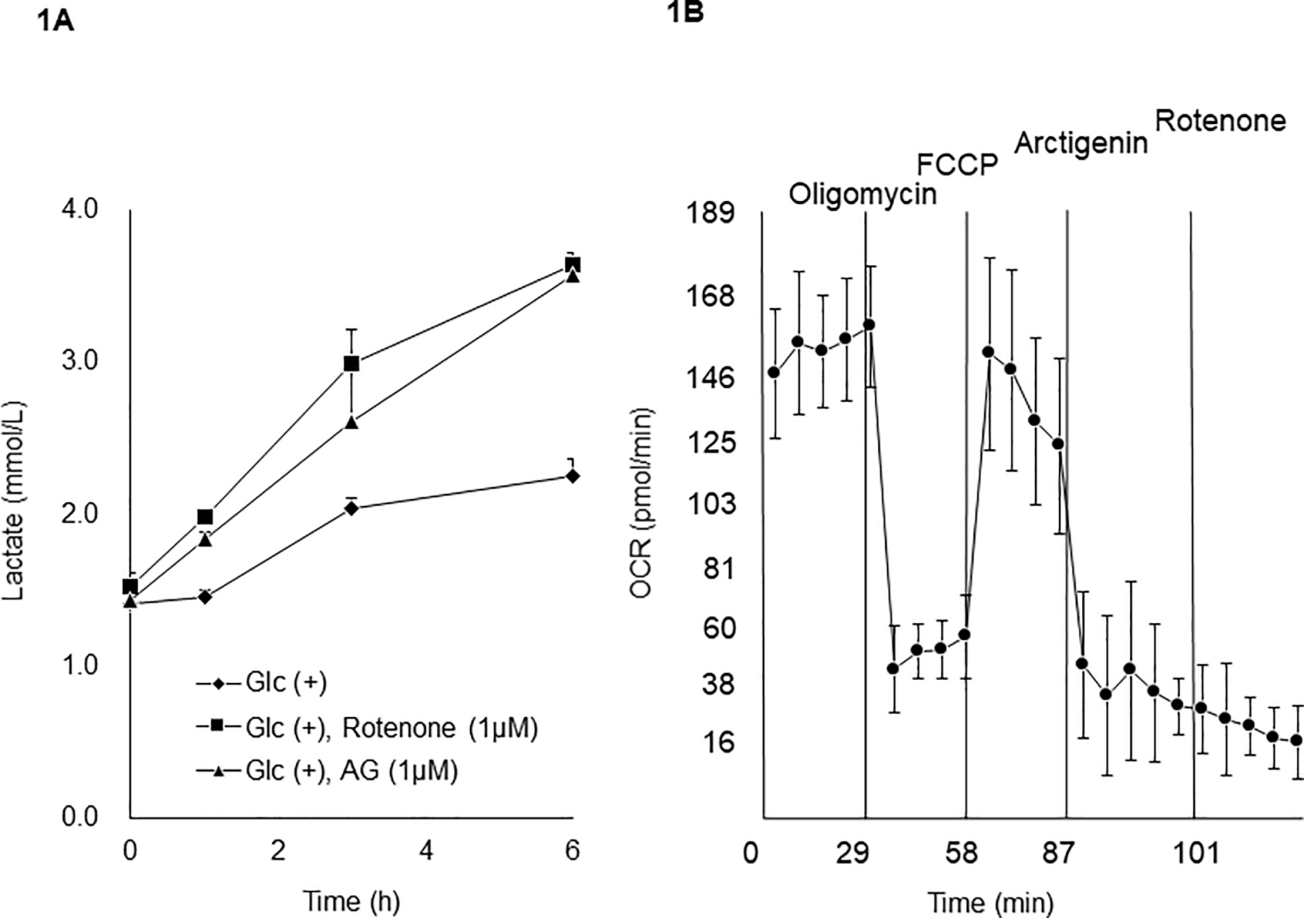
Effect of AG on lactate production by PANC-1 cells and on mitochondrial respiration. A. Lactate concentration in culture media in the absence and presence of AG at 1 μM. Lactate concentration in the medium was sequentially determined by lactate assay kit II. B. Effect of AG on OCR of PANC-1 cells was examined with a Seahorse Extracellular Flux Analyzer model 24XF. Mitochondrial stress test was carried out by inhibiting complex V activity by 1 μM oligomycin, and maximizing respiration was maximized by addition of 1 μM Carbonyl cyanide-p- trifluoromethoxyphenylhydrazone (FCCP). Effect of AG was then examined on the maximized respiration.

When human pancreatic cancer cells PANC-1 were treated with 1 μM AG in culture, a marked increase in lactate concentrations in culture medium was observed (Fig 1A). When OCR was measured with a flux analyzer, clear inhibition of OCR was observed at 1 μM AG (Fig 1B). These observations were consistent with those in a previous report [16] and suggested that lactate concentrations is increased in plasma of patients administered GBS-01 containing 100–400 mg AG, depending on the dose of GBS-01 administered in the phase I clinical trial.

### Plasma lactate concentrations in patients administered GBS-01

**Fig 2:**
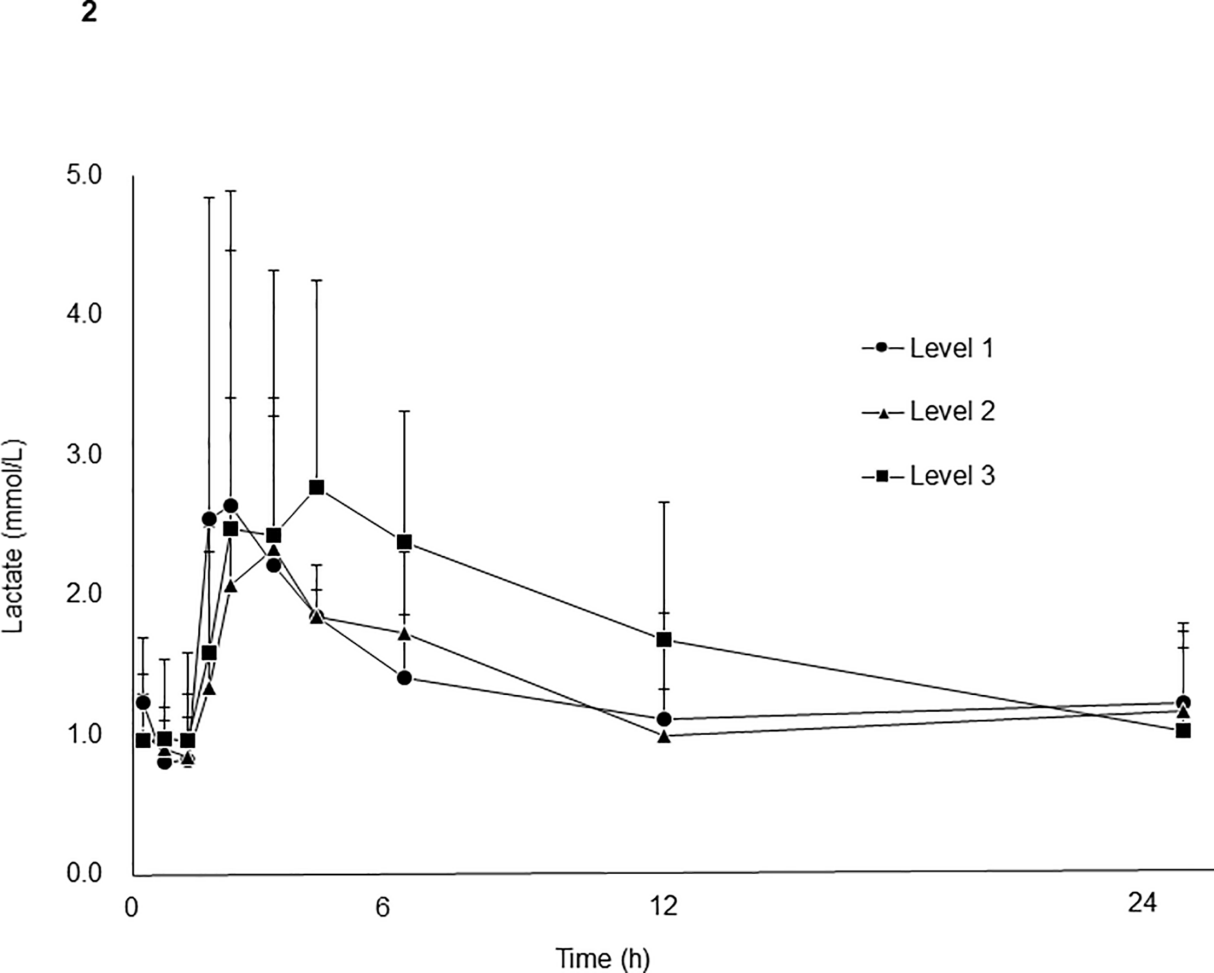
Time-dependent change in plasma lactate in patients in GBS-01 phase I clinical trial (UMIN000005787). Patients was orally administrated GBS-01 corresponding to 100 mg (level 1), 250mg (level 2) and 400mg (level 3) equivalent of arctigenin. Number of patients were 3, 3 and 9 in level 1, level 2, and level 3, respectively. Each point represents mean and arrows represent standard deviation.

Plasma samples from patients enrolled in a phase I clinical trial of GBS-01 were analyzed for lactate concentrations. In the phase I trial, patients were administered three different doses of GBS-01: 3, 7.5, and 12 g of GBS-01 containing 100, 250, and 400 mg, respectively, of AG equivalent. The mean plasma lactate concentrations in patients receiving each level of GBS-01 dose are plotted in Fig 2.

AG and AGG concentrations were also analyzed. Cmax and AUC of plasma AG and AGG were well correlated with the dose of GBS-01 administered (data not shown). However, no dose-dependent increase in lactate concentrations or AUC in plasma was observed. A slight increase in lactate AUC in level 3 patients was observed (Fig 2), but it was not statistically significant.

### Plasma lactate concentration and AUC differed among patients

**Fig 3:**
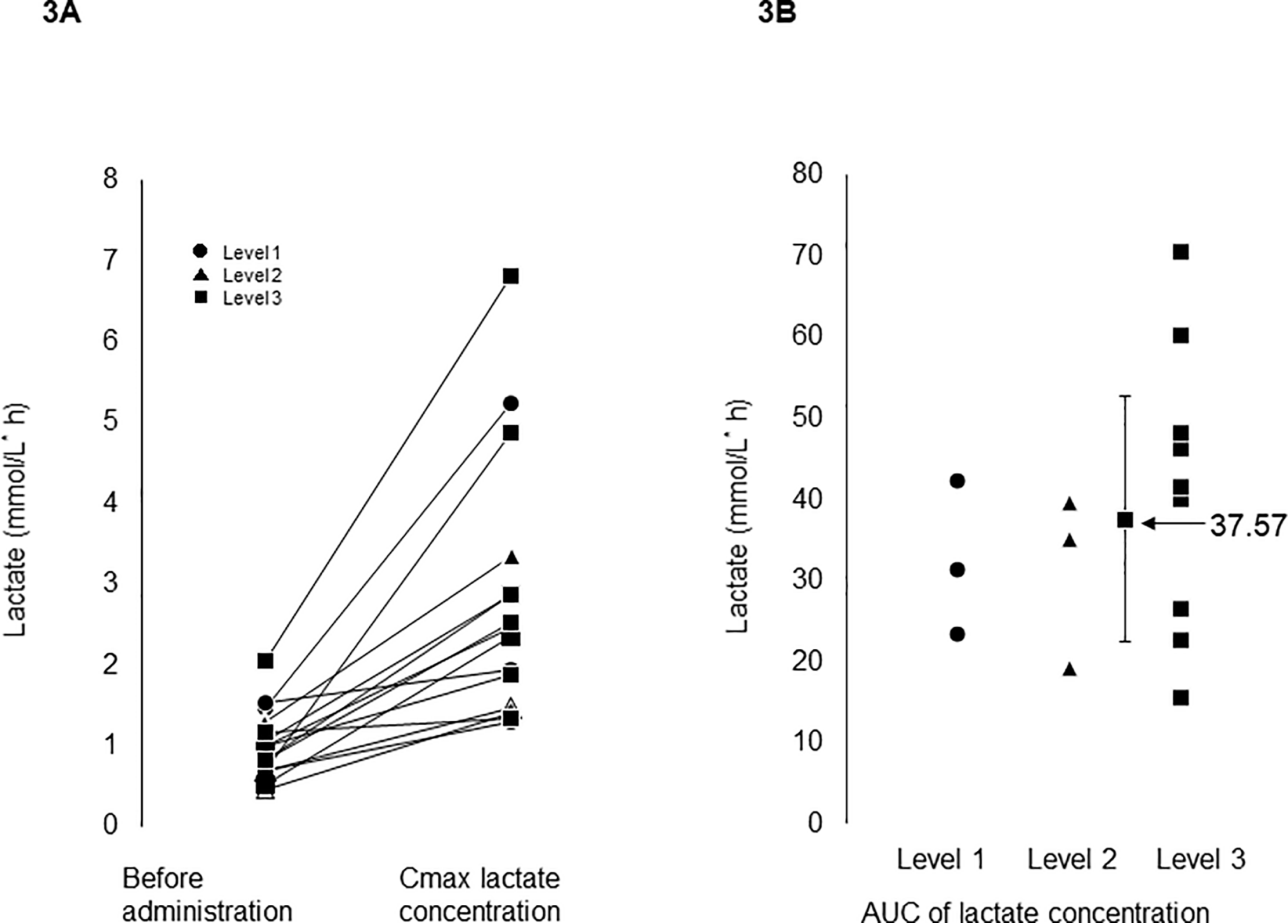
Individual variation of plasma lactate maximal concentration and plasma lactate AUC. A, Correlative plots of plasma lactate level before and after AG administration. Cmax of lactate was used as lactate concentration after AG administration. B, Lactate AUC was plotted.

**Fig 4:**
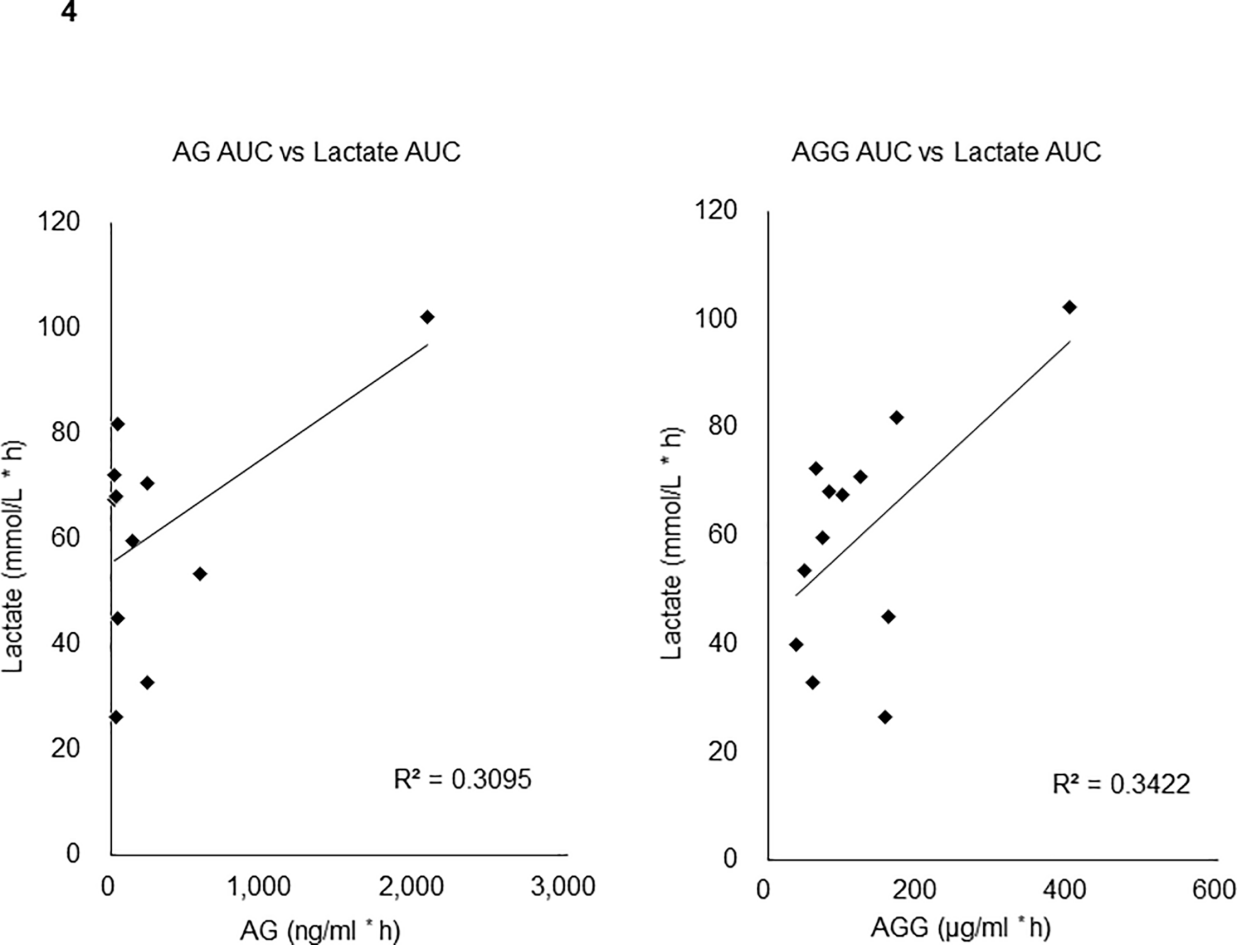
Correlation analyses of plasma lactate AUC and AUC of AG or AGG.

As clearly shown by the large standard deviation in plasma lactate concentrations, individual variation in patients in the same level of GBS-01 was large (Fig 2). In some patients, plasma lactate concentrations did not change after GBS-01 administration, but in others it increased significantly (Fig 3A). This large variation in plasma lactate concentrations was also evident when AUC of the lactate concentration time course was analyzed (Fig 3B). Variation was evident among patients at the same level of GBS-01 administration rather than among levels. No significant correlation was observed between AUCs of plasma lactate and AG and AGG (Fig 4). No significant correlation was observed when Cmax instead of AUC was used for both lactate and AG and AGG (S1 Fig). One healthy volunteer received level 3 GBS-01 and the same pharmacokinetic study and lactate analysis were performed. The results clearly showed a marked increase in AG and AGG concentration after GBS-01 intake, but no increase in lactate concentration was observed (S2 Fig).

### Correlation of plasma lactate and clinical responses

Although plasma lactate Cmax or AUC did not correlated with plasma AG or AGG concentration, there is a possibility that plasma lactate might be a pharmacodynamic biomarker. Regarding to clinical response, clear tumor shrinkage (PR) was observed in one among 15 patients enrolled in the phase I study, long SD was observed in one and SD in three patients.

Seven patients were received more than one couse (28days) of GBS-01 administration but disease progression was observed within the following 28 days (PD) and clinical response was not evaluated in three patients patients because administration was ceased before one course completion [15]. Association between lactate AUC or Cmax and progression free survival time and overall survival of patients was analyzed by Kaplan-Meiyer methods among of lactate high and low groups that were divided in to two by median of Cmax or AUC, but no significant difference was observed in two groups either in terms of progression free survival time or overall survival time (S3 Fig). Statistical difference was also analyzed mean values of Cmax and AUC of lactate between patients showed PD and patients showed PR or SD by Student t-test, but no difference was observed neither in Cmax nor AUC.

### Correlation of plasma lactate and renal and liver function

**Fig 5:**
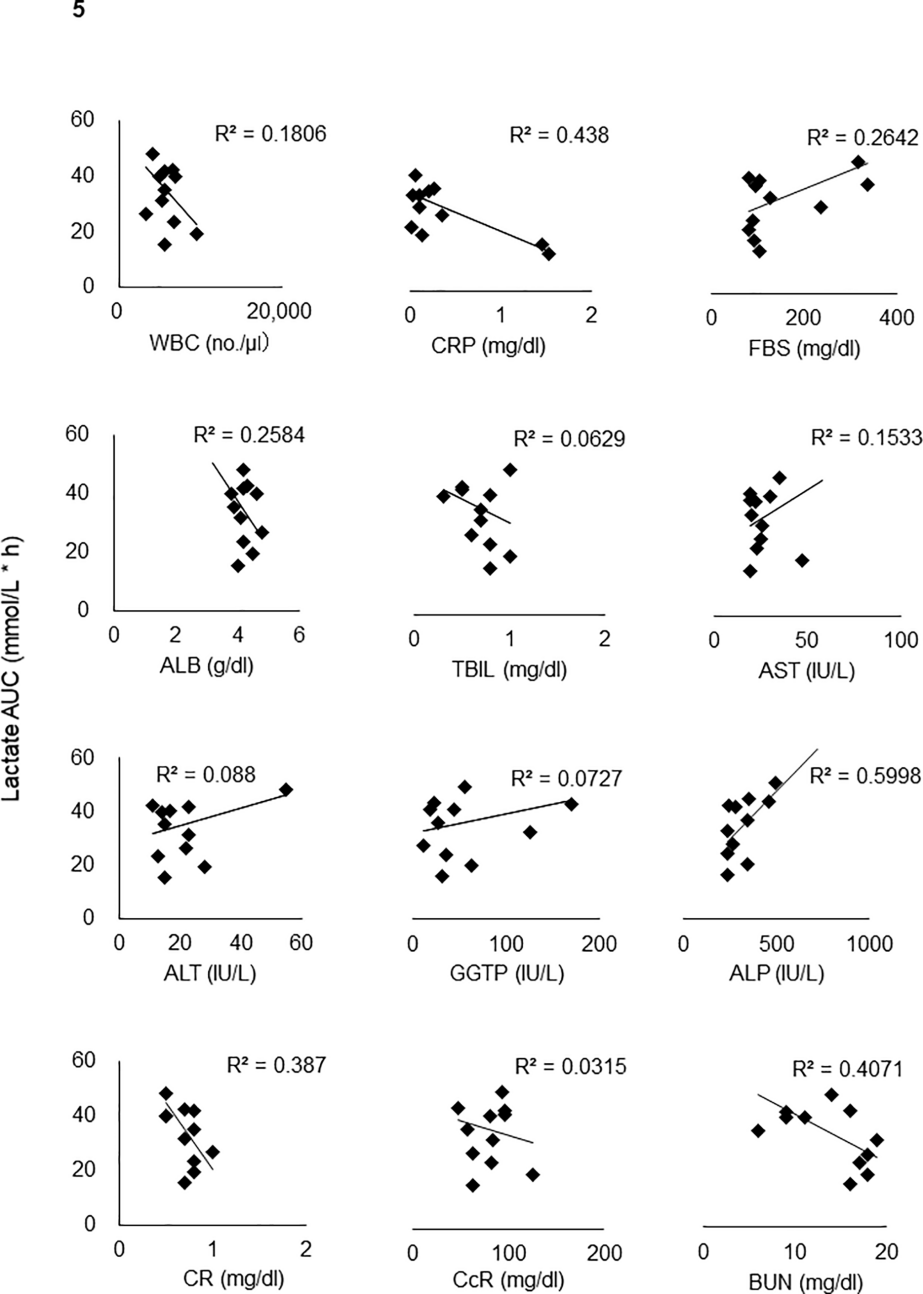
Correlation analyses among plasma lactate AUC and laboratory tests. WBC, CRP, FBS, ALB, T-BIL, AST, ALT, γGTP, ALP, CRE, CCr and BUN.

Patients were enrolled in the study under eligibility criteria including renal and liver function tests, renal and liver function were not seriously impaired in any patients and only minor disorders were noticed. Because no association between plasma AG and lactae was observed and no lactate increase was observed in one healthy volunteer despite good bioavailability, lactate clearance capacity of each patients was suspected to be a determinant of plasma lactate level. Individual Cmax and AUC of plasma lactate were analyzed against the results of the following laboratory tests. As a renal function test, BUN and CRE and as a liver function test, ALB, AST, ALT, ALP, and TBIL were examined. Majority of the results of these function tests showed no significant correlations, but as shown in Fig 5, Table 1, and S1 Table, the liver function tests for ALB and AST showed marginally significant correlations with lactate Cmax and AUC. Multivariate analysis was carried out using SAS among 15 cases and only ALB and AST were slightly correlated with plasma lactate level.

**Table 1:** Multivariate analysis between AUC of lactate and renal and liver function in 15 patients.

	Univariate		Multivariate	
Variable	Estimate	p-Value	Estimate	p-Value
ALB	-18.11 ± 7.73	0.04	-16.52 ± 6.15	0.02
AST	0.30 ± 0.26	0.27	0.41 ± 0.23	0.10
CR	-48.28 ± 26.14	0.09	-33.68 ± 23.5	0.18
BUN	-2.23 ± 0.74	0.01	-1.41 ± 1.01	0.19
GGTP	0.02 ± 0.03	0.51	0.04 ± 0.03	0.20
TBIL	-2.37 ± 12.00	0.85	4.39 ± 11.39	0.71
ALT	-0.03 ± 0.19	0.89	0.03 ± 0.19	0.86
ALP	0.00 ± 0.01	0.74	0.00 ± 0.01	0.95

Based on a multivariate analysis adjusting two possible confounding variables (surgical history and level of GBS-01 dose).

## Discussion

AG has recently been found to have inhibitory activity on mitochondrial complex I. As indicated for metformin and phenformin, lactate production may cause a serious adverse effect when complex I activity is effectively inhibited throughout the body [17]. AG caused a marked increase in lactate accumulation in *in vitro* cell culture. However, in the present study, only a mild and transient increase in plasma lactate was observed. In some patients and one healthy volunteer, no plasma lactate increase was observed. Plasma lactate level is determined by both production rate and clearance rate. Plasma lactate is used for glucose synthesis in the liver by gluconeogenesis in the Cori cycle, and because of this mechanism, lactate increase after vigorous muscle contraction is only transient, although it reaches a maximal 10–15 mM plasma level, without serious condition [19,20].

AG is effectively absorbed from the intestine, and approximately 50% of orally administered AG can be recovered in the urine in glucuronic acid conjugated form in a median time of 24 hours. AG is thus efficiently absorbed in the body, but it rapidly undergoes glucuronic acid conjugation in the liver mainly by UGT1A1 and 1A9 (Kawashima et al., to be published). AGG shows no cytotoxicity during glucose deprivation nor complex I inhibition at the cellular level and is reactivated by cleaving with β-glucuronidase (our unpublished data). Because most of the AG absorbed is rapidly conjugated with glucuronic acid at the first pass in the liver, it is inactivated unless otherwise cleaved by β-glucuronidase in the organs and tissues. It is thus plausible that most of the lactate is produced in the intestine or in the liver before conjugation, but this speculation is difficult to confirm biochemically in the clinical setting. We have examined the possibility of lactate production in the small intestine in a mouse model and the results indicated that most of the lactate is produced in the liver in mouse model. This might be also the case in human.

In the present study, we observed indivisual variation in plasma lactate level. Bioavailability of AG and its stability and absorption in the intestine may differ among individuals. However, difference in bioavailability might not the cases of variation of lactate levels among patients because no positive relationship between plasma AG and lactate was observes.

Another possibility is the rate of lactate reutilization by gluconeogenesis as in the Cori cycle. The Cori cycle is a biochemical process of synthesis of glucose using lactate usually produced in skeletal muscle. A similar biochemical reaction, the alanine–pyruvate cycle [21] is also a safeguard against lactate acidosis. These biochemical processes primarily depend on the liver function and the metabolic status of the liver. Gluconeogenesis generally operates in the liver under the control of glucagon and insulin. Insulin is inhibitory to gluconeogenesis, including the Cori cycle [22]. Pancreatic cancer patients often suffer from diabetes owing to the destruction of pancreas and to the release of diabetic cytokines from inflammatory cells [23], but this point needs to be analyzed in the future study (S2 Table). Metabolic disturbance in cancer may be associated with increased lactate concentration. Our results indicate that some liver function test results are correlated with the lactate level. The present study was carried out as a phase I study, only patiets with normal liver function were enrolled and this might be the reason for marginal association between lactate level and liver function test. Once AG is administered in a wide variety of patients, liver function needs to be carefully examined in relation to plasma lactate level.

Another possible reason for the variation is difference in reactivation of AGG by β-glucuronidase in peripheral tissues, such as skeletal muscle, kidney, lung, spleen, and pancreas. Once AGG is reactivated, lactate can be produced in that tissue and this lactate is transferred to the liver as in the Cori cycle. β-Glucuronidase is expressed in various organs and tissues. Furthermore, individual variation among patients may be caused mainly by the difference in reactivation of glucuronides and the ability of the liver to clear plasma lactate during gluconeogenesis. β-glucuronidase is expressed in various tissues and cells and sometimes induced by inflammatory cytokines such as Tumor Necrosis Factor (TNF-α) [24].

In the present phase I study, marked antitumor activity of AG was observed in one patient (PR) and SD was observed in four patients. Because of large indivisual valiation of plasma lactate, we expected that plasma lactate might be a biomarker of clinical effectiveness but this was not confirmed in the present study. Clearance of lactate from plasma rather than production rate in organs and tissues might be stronger determinant of plasma lactate level in whole body. Considering the effective *in vitro* AG dose and the plasma AG concentration in these patients, reactivation of glucuronide in the tissues may be an important factor in determining clinical response and lactate production. Antitumor effect by AG exerts locally but not systemically after reactivation by the tumor tissue and this might be the reason why plasma lactate level did not correlate with antitumor effect. More precise study is needed to answer this question.

## Supporting information

S1 Fig Correlation analysis between AG Cmax vs lactate Cmax and AGG Cmax vs lactate Cmax. (TIF)

S2 Fig Time-dependent change in plasma lactate level after GBS-01 administration in a healthy volunteer. A volunteer was received 400mg AG equivalent GBS-01.
(TIF)

S3 Fig Kaplan-Meier analysis of patients enrolled in GBS-01 phase I clinical trial. Patients were stratified into two groups based on plasma lactate level and the median was used to stratify.
(TIF)

S1 Table Multivariate analysis of Cmax of lactate and renal and liver function in 15 patients. (TIF)

S2 Table Information on diabetic condition of 15 patients. (TIF)
