# Supplementary figures and images for: Change in plasma lactate concentration during arctigenin administration in a phase I clinical trial in patients with gemcitabine-refractory pancreatic cancer
Source: PLoS One. 2018 Jun 1;13(6):e0198219. doi: 10.1371/journal.pone.0198219 (PMC5983509; doi:10.1371/journal.pone.0198219)

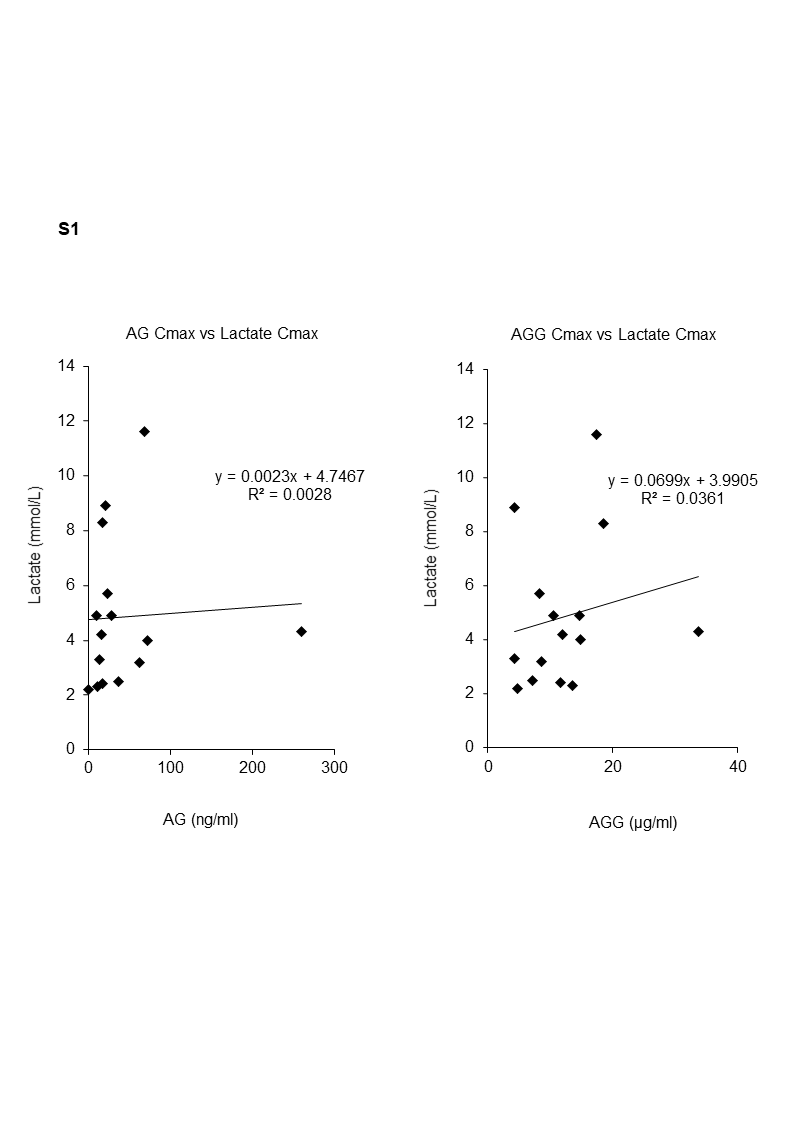

Supplement: S1 Fig — (TIF) [file pone.0198219.s001.tif]

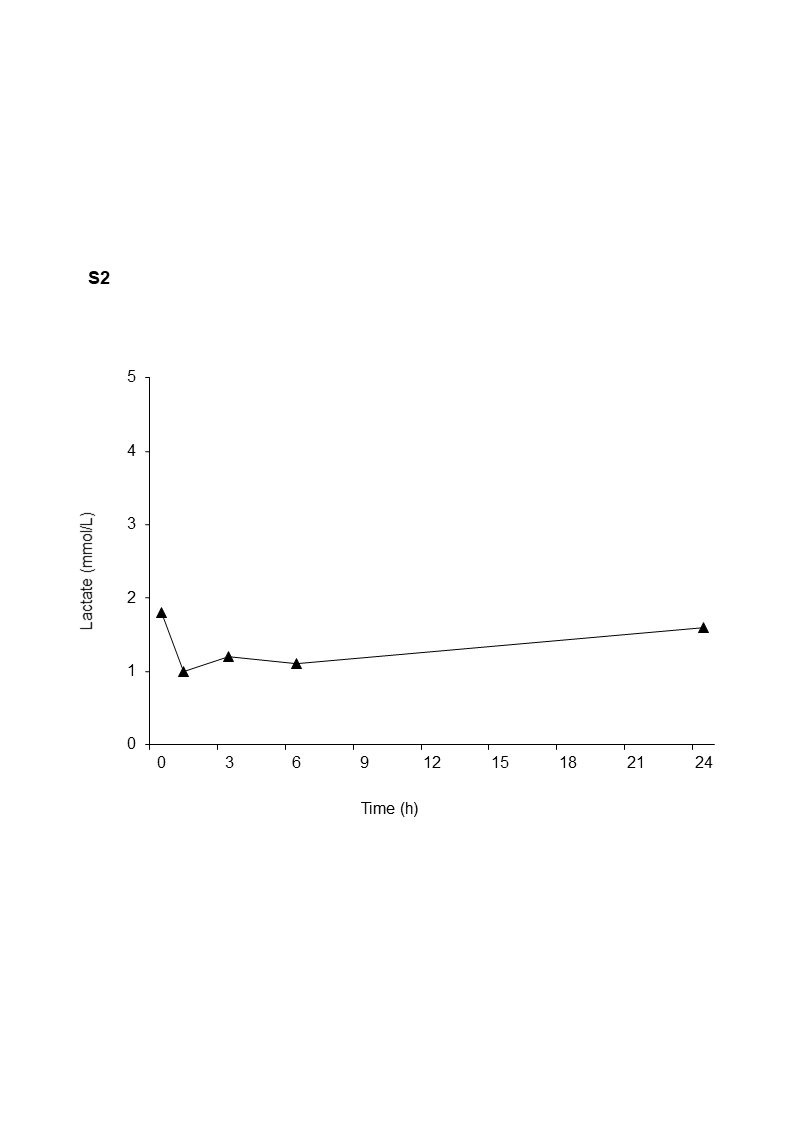

Supplement: S2 Fig — A volunteer was received 400mg AG equivalent GBS-01. (TIF) [file pone.0198219.s002.tif]

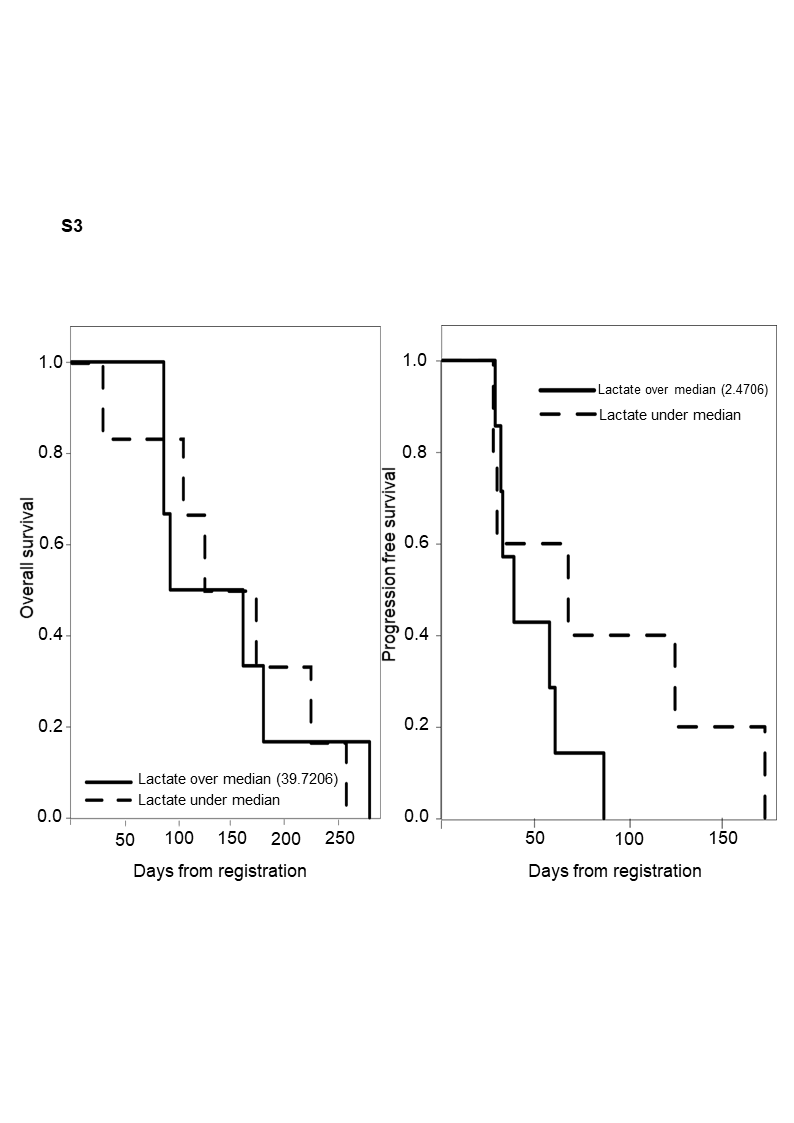

Supplement: S3 Fig — Patients were stratified into two groups based on plasma lactate level and the median was used to stratify. (TIF) [file pone.0198219.s003.tif]

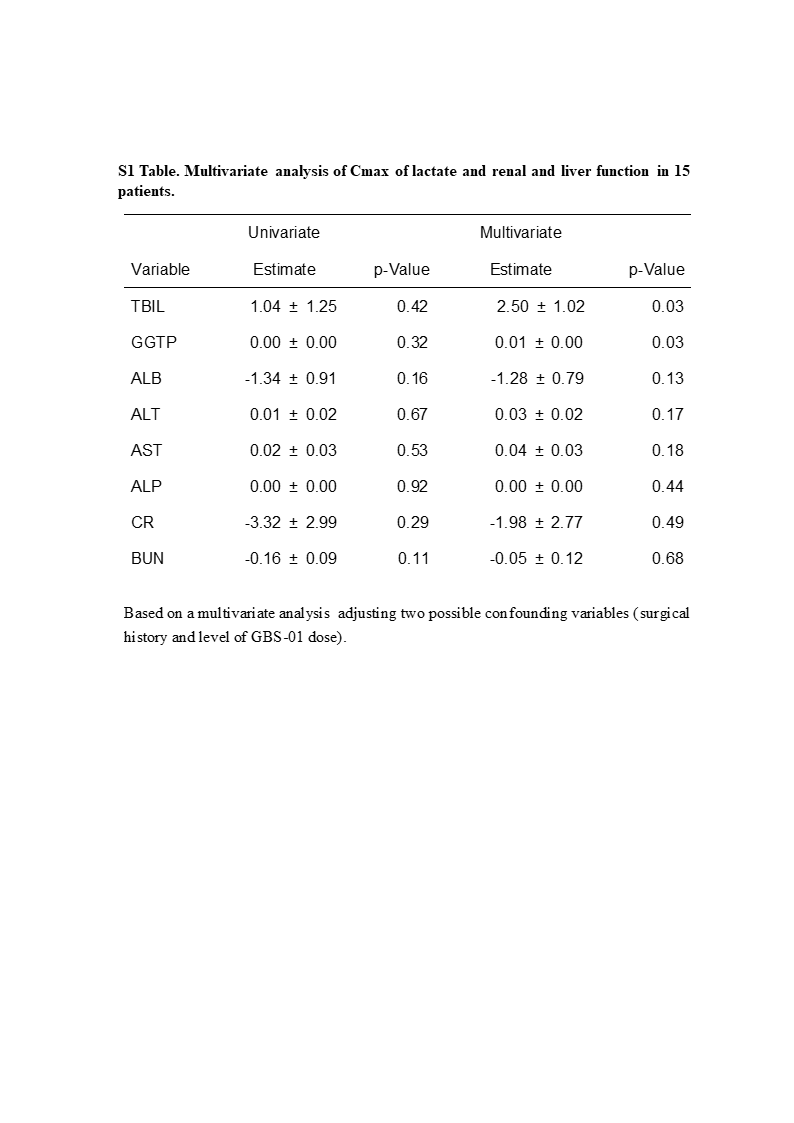

Supplement: S1 Table — (TIF) [file pone.0198219.s004.tif]

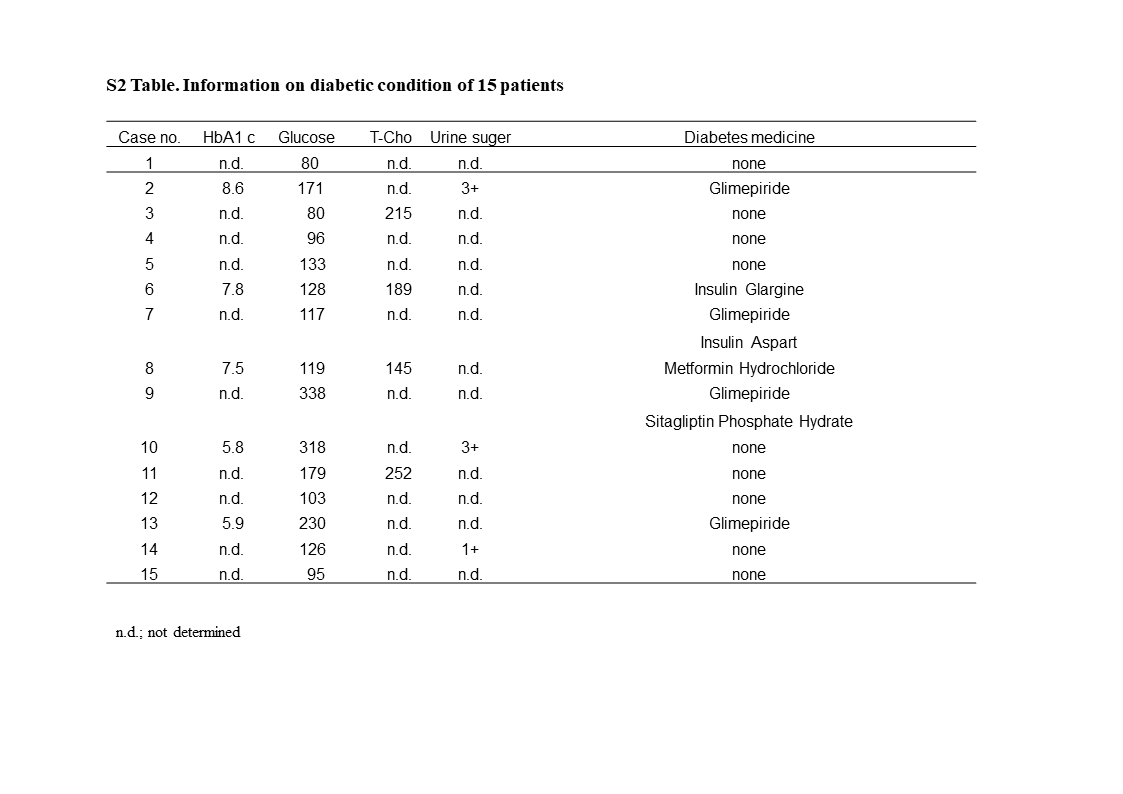

Supplement: S2 Table — (TIF) [file pone.0198219.s005.tif]
